# Identification of Potential Long Non-coding RNA Expression Quantitative Trait Methylations in Lung Adenocarcinoma and Lung Squamous Carcinoma

**DOI:** 10.3389/fgene.2020.602035

**Published:** 2020-12-09

**Authors:** Xiaohong Wu, Yue Gao, Jianlong Bu, Lin Deng, Pinyi Zhang, Meng Chi, Lihua Jiang, Xiaoding Shi, Shangwei Ning, Guonian Wang

**Affiliations:** ^1^Department of Anesthesiology, Harbin Medical University Cancer Hospital, Harbin, China; ^2^College of Bioinformatics Science and Technology, Harbin Medical University, Harbin, China; ^3^Department of Thoracic Surgery, Harbin Medical University Cancer Hospital, Harbin, China

**Keywords:** lung adenocarcinoma, lung squamous carcinoma, lncRNAs, methylation, prognostic biomarker, functional analysis

## Abstract

There are associations between DNA methylation and the expression of long non-coding RNA (lncRNA), also known as lncRNA expression quantitative trait methylations (lnc-eQTMs). Lnc-eQTMs may induce a wide range of carcinogenesis pathways. However, lnc-eQTMs have not been globally identified and studied, and their roles in lung adenocarcinoma (LUAD) and lung squamous carcinoma (LUSC) are largely unknown. In the present study, we identified some differential methylation sites located in genes of long intergenic non-coding RNAs (lincRNAs) and other types of lncRNAs in LUAD and LUSC. An integrated pipeline was established to construct two global cancer-specific regulatory networks of lnc-eQTMs in LUAD and LUSC. The associations between eQTMs showed common and specific features between LUAD and LUSC. Some lnc-eQTMs were also related with survival in LUAD- and LUSC-specific regulatory networks. Lnc-eQTMs were associated with cancer-related functions, such as lung epithelium development and vasculogenesis by functional analysis. Drug repurposing analysis revealed that these lnc-eQTMs may mediate the effects of some anesthesia-related drugs in LUAD and LUSC. In summary, the present study elucidates the roles of lnc-eQTMs in LUAD and LUSC, which could improve our understanding of lung cancer pathogenesis and facilitate treatment.

## Introduction

Lung cancer is one of the major causes of cancer-related death globally (Saracci and Wild, 2016). Lung cancer accounts for 19% of cancer deaths worldwide and 3% of all deaths (Cryer and Thorley, 2019). In addition, lung cancer has a dismal 5 year survival rate of approximately 19%, second only to pancreatic cancer, the cancer with the poorest prognosis (Siegel et al., 2020). Of all lung cancer cases, non-small cell lung cancer (NSCLC) is the predominant pathological subtype, accounting for approximately 85% (Rotow and Bivona, 2017; Terlizzi et al., 2019). NSCLC includes lung squamous cell carcinoma (LUSC), lung adenocarcinoma (LUAD), and large cell carcinoma. Targeted drugs are available for a certain number of patients with NSCLC harboring gene alterations. Although targeted drugs significantly improve survival compared with chemotherapeutic drugs alone, the difference between LUAD and LUSC remains to be analyzed at the molecular level (Park and Han, 2019).

The associations between protein-coding genes and lung cancer have been a hot study topic for several decades. Long non-coding RNAs (lncRNAs), which is considered as a type of common non-coding RNAs could play important roles in several biological processed including cancer occurrence and development (Gugnoni and Ciarrocchi, 2019; Lorenzi et al., 2019). Recently, the dysregulation of lncRNAs in many kinds of cancer, including LUAD and LUSC, has been well-recognized. For example, lncRNA TINCR is downregulated in LUAD and LUSC tissues compared with normal tissues (Wei and Zhang, 2016). LncRNA SNHG1 is upregulated in LUSC tissues. Knockdown of SNHG1 significantly inhibits the proliferation, metastasis, and invasive ability of LUSC cells and induces apoptosis. The relationships between numerous lncRNAs and lung cancer have been reported, but the biological functions and underlying mechanisms of only a handful of them are well-characterized. In particular, little is known about the difference between LUAD and LUSC at the lncRNA level.

DNA methylation is a chemical DNA modification which can change the genetic expression without changing the DNA sequence. DNA methylation refers to the covalent binding of a methyl group to the C_5_ atom of cytosine and is involved in several physiological processes and pathologic conditions, such as cancer (Hamidi et al., 2015; Schubeler, 2015). For instance, methylation of H3K36 activates NOTCH signaling to drive breast tumor initiation and metastatic progression (Jeong et al., 2020). Functional interplay may take place between DNA methylation and lncRNA expression, because both of them are regulated dependently on developmental stage and cell type (Yuan et al., 2016). In fact, accumulating evidence has uncovered the underlying crosstalk between lncRNAs and the methylation regulatory network. Many earlier studies reported significant associations between DNA methylation and lncRNA expression [lncRNA expression quantitative trait methylations (lnc-eQTMs)]. For example, knockdown of lncRNA HOTAIR has been carried out in multidrug-resistant lung cancer cell lines (H69AR and H446AR) to assess its influence on chemoresistance. Depletion of HOTAIR weakens HOXA1 methylation by decreasing DNMT1 and DNMT3b expression and participates in chemoresistance in lung cancer (Fang et al., 2016). In view of the considerable diversity with respect to the relationships between lncRNAs and DNA methylation in important biological processes, some research methods specific for lncRNAs and methylation have been developed (Zhi et al., 2018; Gao et al., 2019). However, systematic knowledge and mechanism on regulatory lncRNA–methylation relationships in LUAD and LUSC is still lacking.

In the present study, some differential methylation sites (DMSs) located in genes encoding long intergenic non-coding RNAs (lincRNAs) and other types of lncRNAs were identified in LUAD and LUSC. Two global cancer-specific regulatory networks of lnc-eQTMs in LUAD and LUSC were constructed based on an integrated computational pipeline. The lncRNAs in these two lnc-eQTM networks were considered as DMS-mediated lncRNAs (DMSmlncRNAs). The associations between eQTMs showed common and specific features between LUAD and LUSC. Some DMSmlncRNAs in LUAD- and LUSC-specific regulatory networks were related with survival. Functional analyses showed that these DMSmlncRNAs were associated with cancer-related functions, such as lung epithelium development and vasculogenesis. Drug repurposing analysis found that these DMSmlncRNAs may mediate the effects of some anesthesia-related drugs in LUAD and LUSC. Collectively, this study elucidates the function of DMSs and DMSmlncRNA in LUAD and LUSC.

## Materials and Methods

### Collecting High-Throughput Expression and Clinical Information of LncRNAs in LUAD and LUSC

Large-scale lncRNA expression profiles (Illumina HiSeq level 3) and clinical follow-up survival time of LUAD and LUSC patients were obtained from The Cancer Genome Atlas (TCGA) data portal (https://xenabrowser.net/datapages/; release: 2017-09-08). The normal control samples of LUAD and LUSC were included in our analysis. The lncRNAs with expression values of 0 in all samples were excluded. The lncRNAs with expression values of 0 in all samples were excluded. In this way, 15,364 (96.76%) and 15,366 (96.78%) lncRNAs are selected in LUAD and LUSC, respectively, for following analysis. Any remaining expression values of 0 were set to the minimum value of all samples, and all values were log_2_-transformed to obtain a normal distribution. The genome annotation data including genome sites and symbols of lncRNAs were obtained from GENCODE 32 (https://www.gencodegenes.org/; release: 2019-09-19). The lncRNAs were divided into lincRNAs and other lncRNAs, including antisense, transcribed_processed_pseudogene, and sense_overlapping.

### Methylation Profile and Data Processing of LUAD and LUSC

The methylation profiles (level 3) of the Illumina 450k Methylation chip for LUAD, LUSC, and their matched normal tissues were also downloaded from TCGA. Methylation sites with NA in all samples were removed. The methylation sites were mapped to the lncRNAs using BEDTools (V2.29.0)^1^ according to diverse genomic locations in GENCODE.

### Identification of DMSs Located at LncRNAs for LUAD and LUSC

DMSs between lung cancer and corresponding normal samples for a specific methylation site were identified using the two-tailed Student’s *t*-test, with a false discovery rate (FDR) <0.05 and the absolute methylation difference between the mean methylation values of the lung cancer and normal samples >0.3 at the methylation site. DMS refers to a single differentially methylated CpG site.

### Construction of LUAD- and LUSC-Specific Lnc-eQTM Networks

An integrated computational pipeline was constructed to identify LUAD- and LUSC-specific lnc-eQTM networks. First, only DMSs and their corresponding lncRNAs were extracted to construct networks. Second, Pearson correlation coefficients (PCCs) were calculated between methylation levels and lncRNA expression in LUAD and LUSC and normal control samples. Third, absolute difference values of PCCs between lung cancer and normal samples were also obtained. Lastly, the associations would be considered as lung cancer-specific methylation–lncRNA interactions if the absolute difference values were larger than 0.3. The lncRNAs in these associations were considered as DMSmlncRNAs. The networks were constructed by Cytoscape 3.3.0^2^.

### Calculating Integrated Risk Score and Survival Analysis in LUAD and LUSC

In order to evaluate the ability of predicting survival for each lnc-eQTM, a comprehensive computational approach was developed based on survival, lncRNA expression, and methylation data. A multivariate Cox regression model was established for the methylations related to the same DMSmlncRNA in a specific lnc-eQTM. The integrated risk score for each lung cancer patient was calculated according to the linear combination of the lncRNA expression values weighted by the coefficient from multivariate Cox regression analysis as follows:

$$RiskScore=\sum_{i=1}^{n}cox_{i}*Methylation\left(meth_{i}\right)$$

where *cox*_*i*_ is the Cox regression coefficient of the *i*th methylation site and *n* is the number of methylations regulated by the same DMSmlncRNA. Methylation (*meth*_*i*_) is the methylation level of the *i*th methylation site in the LUAD and LUSC patients. The LUAD and LUSC patients were divided into high and low risk groups based on median values of the risk score. Lastly, we performed Kaplan–Meier survival analysis for these two groups and the log-rank test was used to evaluate statistical significance. The survival results were considered significant if *P* < 0.05. All analyses were performed within the R 3.3.3 framework.

### Functional and Drug Analyses for Lnc-eQTMs in LUAD and LUSC

We used DMSmlncRNAs in LUAD- and LUSC-specific eQTM networks to perform functional and drug enrichment analyses using Enrichr online tool^3^ (Kuleshov et al., 2016) with default parameters. The Gene Ontology (GO) terms and drugs were extracted if *P*-values were smaller than 0.05.

## Results

### DMSs and Their Corresponding LncRNAs Were Identified in LUAD and LUSC

**FIGURE 1:**
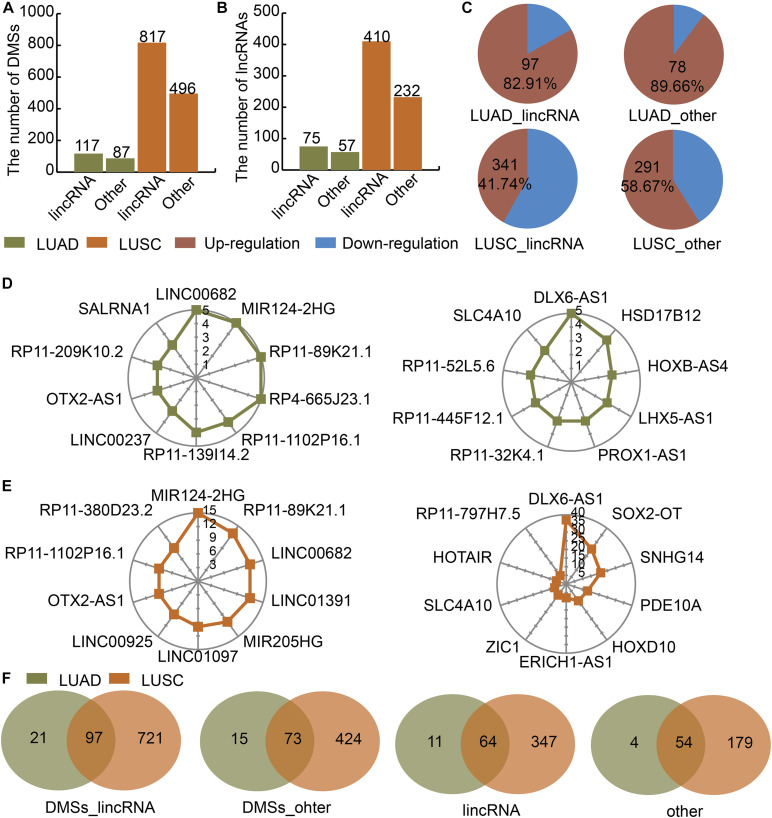
Identification of DMSs and their corresponding DMSmlncRNAs in LUAD and LUSC. **(A)** Barplot showing the number of DMSs in LUAD (green) and LUSC (orange). **(B)** Barplot showing the number of DMSmlncRNAs in LUAD and LUSC. **(C)** Pie charts showing the percentages of up- (red) and downregulated (blue) DMSs in lincRNAs and other lncRNAs in LUAD and LUSC. **(D)** Radar maps showing the top DMSmlncRNAs with the highest numbers of DMSs in LUAD. **(E)** Radar maps showing the top DMSmlncRNAs with the highest numbers of DMSs in LUSC. **(F)** Venn diagrams showing common DMSs in genes encoding lincRNAs and other lncRNAs in LUAD (green) and LUSC (orange).

We divided the lncRNAs into lincRNAs and other types in LUAD and LUSC. Then we identified DMSs in lincRNAs and other type of lncRNAs. There were 117 and 87 DMSs located at lincRNAs and other types of lncRNAs in LUAD, respectively (Figure 1A). There were 817 and 496 DMSs located at lincRNAs and other types of lncRNAs in LUSC, respectively (Figure 1A). The numbers of lincRNAs and other types of lncRNAs containing DMSs were 75 and 57 in LUAD, respectively (Figure 1B). These two numbers were 410 and 232 in LUSC (Figure 1B). The regulation directions of DMSs were also different between LUAD and LUSC. In LUAD, 82.91 and 89.66% of DMSs in lincRNAs and other types of lncRNAs were upregulated, respectively (Figure 1C). In LUSC, 41.74 and 85.67% of DMSs in lincRNAs and other types of lncRNAs were upregulated, respectively (Figure 1C). The number of DMSs for each lncRNA showed an obvious gap. For example, in LUAD, the top lincRNAs containing DMSs were LINC00682, MIR124-2HG, and RP11-89K21.1 in LUAD (Figure 1D), and the top other types of lncRNAs containing DMSs were DLX6-AS1, HSD17B12, and HOXB-AS4 (Figure 1D). The lncRNAs in LUSC showed a similar pattern (Figure 1E). There were large overlaps of DMSs and lncRNAs between LUAD and LUSC (Figure 1F). These results indicate that DMSs located at lncRNAs may play essential roles in LUAD and LUSC.

### LUAD- and LUSC-Specific Lnc-eQTM Networks Showed Specific Features

**FIGURE 2:**
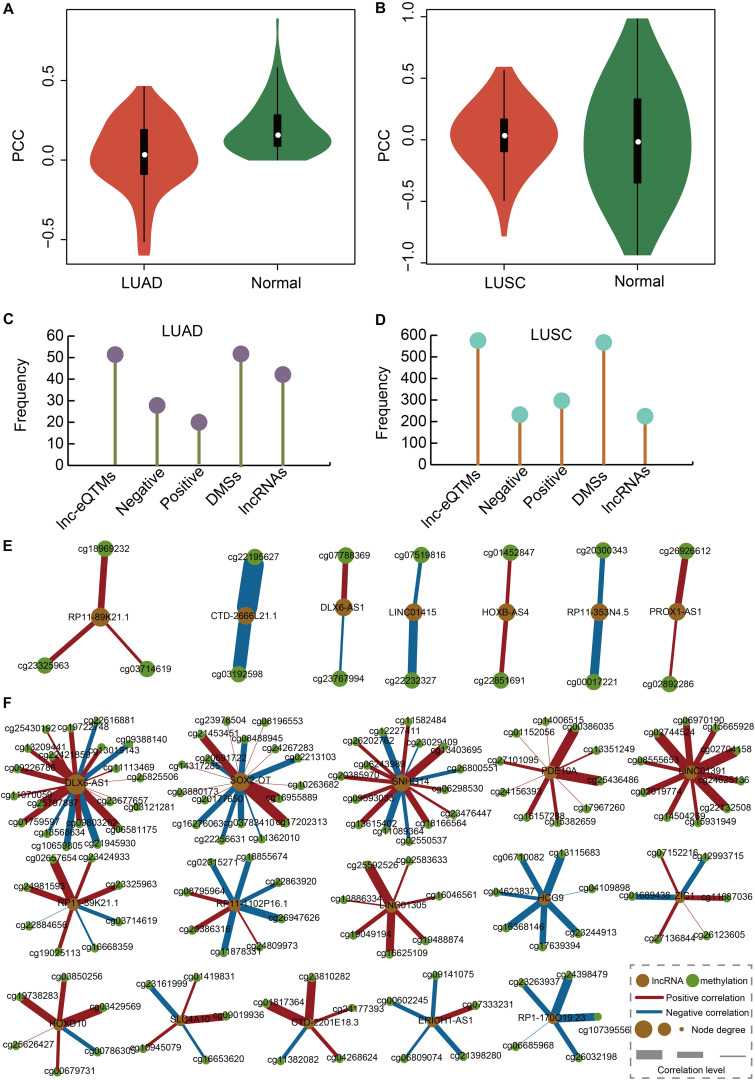
Construction of LUAD- and LUSC-specific lnc-eQTM networks. **(A)** Violin plot showing PCCs between DMSs and corresponding DMSmlncRNAs in LUAD and normal tissues. **(B)** Violin plot showing PCCs between DMSs and corresponding DMSmlncRNAs in LUSC and normal tissues. **(C)** Lollipop graph showing the number of lnc-eQTMs, negative lnc-eQTMs, positive lnc-eQTMs, DMSs, and lncRNAs in LUAD. **(D)** Lollipop graph showing the number of lnc-eQTMs, negative lnc-eQTMs, positive lnc-eQTMs, DMSs, and lncRNAs in LUSC. **(E)** LUAD-specific network. Orange and green represent lncRNA and methylation sites; red and blue edges represent positive and negative interactions; and thicker edges and bigger nodes represent stronger interactions and higher degrees, respectively. **(F)** LUSC-specific network.

We calculated the correlation between DMSs and their corresponding lncRNAs in LUAD, LUAS, and their matched control tissues to identify lnc-eQTMs. We found significant differences with respect to the PCCs between LUAD and normal tissues (Figure 2A). Similarly, there were some differences with respect to the PCCs between LUSC and normal tissues (Figure 2B). Only lnc-eQTMs which showed significant differences with respect to PCCs between lung cancer and normal tissues were considered as lung cancer-specific lnc-eQTMs. In LUAD, 55 specific lnc-eQTMs, including 31 positive and 24 negative interactions, were found in 55 DMSs and 46 lncRNAs (Figure 2C). In LUSC, 593 specific lnc-eQTMs, including 266 positive and 327 negative interactions, were found in 593 DMSs and 241 lncRNAs (Figure 2D). LUAD- and LUSC-specific lnc-eQTM networks were constructed (Figures 2E,F). Regulation directions and regulation intensity were different for each specific lnc-eQTM in LUAD and LUSC. For example, lncRNA DLX6-AS1 and 20 DMSs formed LUSC-specific lnc-eQTMs. For these DLX6-AS1-centric lnc-eQTMs, DMS cg21945930 showed the strongest correlation difference with DLX6-AS1 in LUSC (PCC = 0.14) and normal tissues (PCC = 0.83, PCC difference = 0.69). The correlation between cg21945930 and DLX6-AS1 showed a strong change in LUSC compared with normal tissues. Most DMSmlncRNAs had both up- and downregulated correlations. However, some DMSmlncRNAs, such as LIN01305 and HCG9, only exhibited one direction of regulation.

### The Common and Specific Lnc-eQTMs Between LUAD and LUSC Elucidate Mechanisms Underlying Lung Cancer Subtypes

**FIGURE 3:**
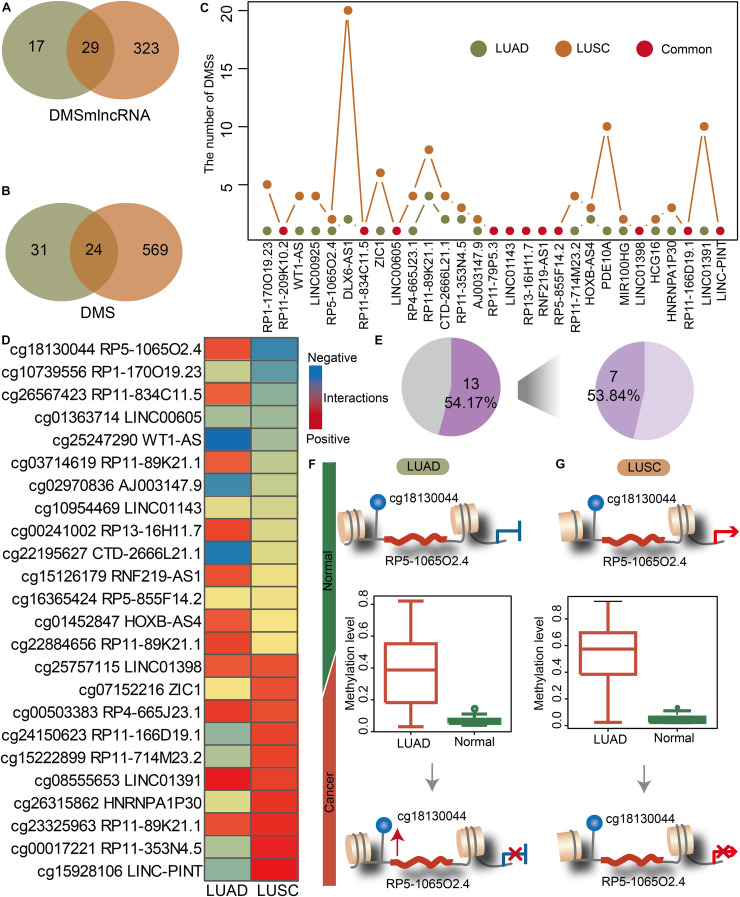
The common and specific lnc-eQTMs between LUAD and LUSC indicate differences in the mechanisms underlying different lung cancer subtypes. **(A)** Venn plot showing common DMSmlncRNAs in lnc-eQTMs between LUAD (green) and LUSC (orange). **(B)** Venn plot showing common DMSs in lnc-eQTMs between LUAD (green) and LUSC (orange). **(C)** Line plot showing the number of DMSs for common DMSmlncRNAs in LUAD and LUSC. **(D)** Heatmap showing the PCCs of each common lnc-eQTM in LUAD and LUSC. Red and blue represent positive and negative interactions, respectively. **(E)** Pie chart showing the percentage of opposite PCCs between LUAD and LUSC. **(F)** The possible mechanism of lnc-eQTMs in LUAD. **(G)** The possible mechanism of lnc-eQTMs in LUSC.

We investigated the lnc-eQTMs’ performance to distinguish lung cancer subtypes. NSCLC mainly includes two major histology subtypes: LUAD and LUSC. Many studies have reported that LUAD and LUSC showed obvious differences in their molecular mechanisms, prompting us to investigate whether specific lnc-eQTMs related to LUAD and LUSC exist. In LUAD- and LUSC-specific lnc-eQTM networks, 29 and 24 common DMSmlncRNAs and DMSs were discovered (Figures 3A,B). Although LUAD and LUSC shared 29 common lncRNAs, most of them contained diverse numbers of DMSs in LUAD- and LUSC-specific lnc-eQTMs (Figure 3C). For example, lncRNA DLX6-AS1 contained 20 DMSs in LUSC and only two DMSs in LUAD. The change level of correlations between DMSs and lncRNAs for 24 common DMSs were also different in LUAD and LUSC (Figure 3D). Of lnc-eQTMs, 54.17% showed opposite regulation directions in LUAD and LUSC (Figure 3E). The lnc-eQTM cg18130044 and RP5-1065O2.4 was considered as an example to explore the functional mechanism of lnc-eQTMs in LUAD and LUSC. In LUAD-matched normal tissues, methylation of cg18130044 could regulate lncRNA RP5-1065O2.4 and inhibit its expression (Figure 3F). Methylation of cg19130044 was upregulated, relieving the inhibition of RP5-1065O2.4 expression in LUAD tissues. However, the mechanism of this lnc-eQTM in LUSC was different. In LUSC-matched normal tissues, methylation of cg18130044 could regulate lncRNA RP5-1065O2.4 and enhance its expression (Figure 3G). The methylation level of cg19130044 was also upregulated, blocking the enhancement of RP5-1065O2.4 expression in LUSC tissues. These results indicate that common lnc-eQTMs may play diverse roles in LUAD and LUSC.

### Some Lnc-eQTMs Were Associated With Survival in LUAD and LUSC

**FIGURE 4:**
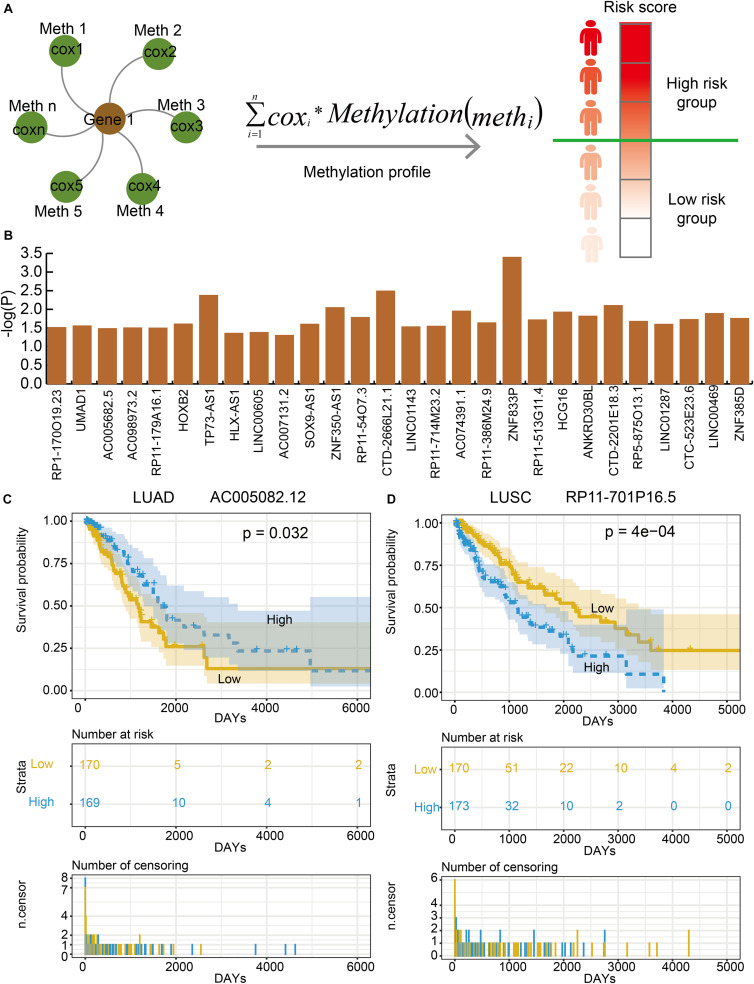
Some lnc-eQTMs were associated with LUAD and LUSC patients’ survival. **(A)** An integrated model for identifying prognosis-related lnc-eQTMs. Firstly, a multivariate Cox regression model was established for the methylations related to the same DMSmlncRNA in a specific lnc-eQTM. Secondly, the integrated risk score for each lung cancer patient was calculated according to the linear combination of the lncRNA expression values weighted by the coefficient from multivariate Cox regression analysis. Thirdly, all the patients were divided to high- and low-risk group for follow survival analysis. **(B)** The *P*-values of prognosis-related lnc-eQTMs in LUSC. **(C)** Survival curves of AC005082.12 in LUAD. **(D)** Survival curves of RP11-701P16.5 in LUSC.

In order to evaluate the effects of lnc-eQTMs on survival in LUAD and LUSC patients, we developed an integrated computational approach to identify prognosis-associated lnc-eQTMs (Figure 4A, see section “Materials and Methods”). For each lnc-eQTM, lncRNA expression and methylation level were integrated into a risk score in LUAD and LUSC samples. The LUAD and LUSC samples were divided into two groups based on integrated risk scores. Lastly, two and 28 prognosis-related lnc-eQTMs were discovered in LUAD and LUSC (Figure 4B). For example, lnc-eQTM AC005082.12 was significantly related with survival in LUAD patients (*P* = 0.032, Figure 4C). Lnc-eQTM RP11-701P16.5 was significantly related with survival in LUSC patients (*P* = 4e–4, Figure 4D). These results indicate that some lnc-eQTMs maybe have the potential to become prognostic biomarkers for LUAD and LUSC.

### LUAD- and LUSC-Specific Lnc-eQTMs Were Related to Essential Functions and Some Drugs

**FIGURE 5:**
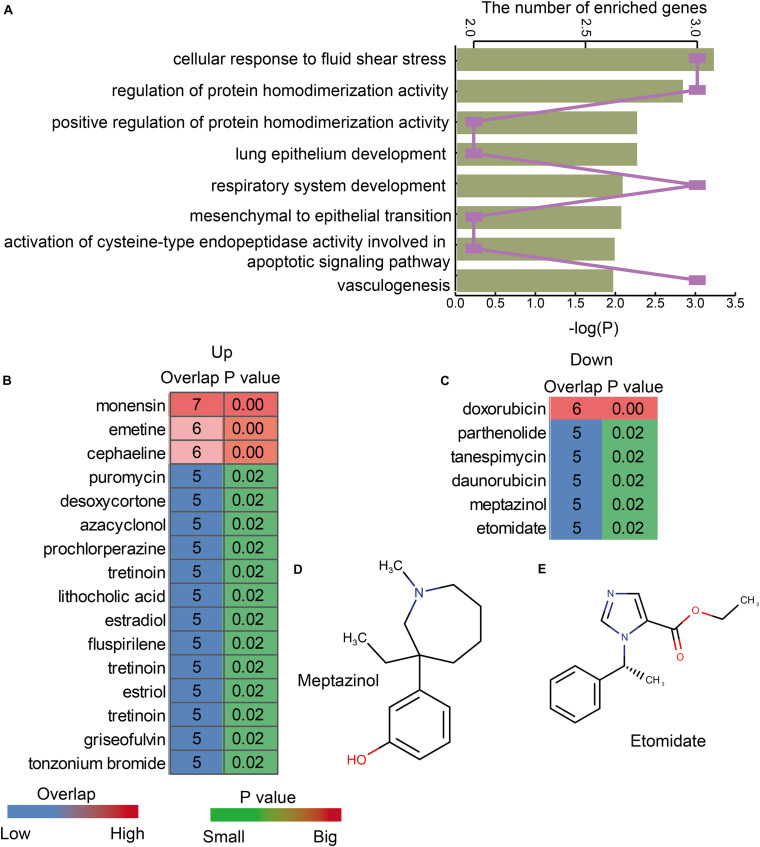
LUAD- and LUSC-specific lnc-eQTMs were associated with some essential functions and drugs. **(A)** Bar chart showing the *P*-values of enriched GO terms and line chart showing the number of enriched genes. **(B)** Heatmap showing the upregulated drugs associated with lnc-eQTMs. **(C)** Heatmap showing the downregulated drugs associated with lnc-eQTMs. **(D)** The chemical constitution of Meptazinol. **(E)** The chemical constitution of Etomidate.

In order to explore the functions of lnc-eQTMs in LUAD and LUSC, functional analysis was performed for lncRNAs in all LUAD- and LUSC-specific lnc-eQTMs. We found these lncRNAs were enriched in some essential functions, such as cellular response to fluid shear stress, lung epithelium development, and vasculogenesis (Figure 5A). Summer et al. (2019) uncovered that the mitochondrial biogenesis pathway driven by the mTOR/PGC1 axis was significantly upregulated in senescent lung epithelial cells. Senescence was originally thought to be a protective mechanism of cancer (Summer et al., 2019). Vasculogenesis plays a major role during tumor growth, and growth of solid tumors beyond 1–2 mm in diameter requires the induction of new blood vessel formation (Kaessmeyer et al., 2014). These DMSmlncRNAs were also associated with some common drugs. The upregulated DMSmlncRNAs were related to some anti-cancer drugs, such as puromycin (Figure 5B), and some sedatives and hypnotic drugs, such as prochlorperazine and fluspirilene. The downregulated DMSmlncRNAs were also related to some anti-cancer drugs, such as doxorubicin, tanespimycin, and daunorubicin (Figure 5C), and some sedative-related drugs, such as Meptazinol (Figure 5D) and Etomidate (Figure 5E). Etomidate was considered as an imidazole derivative with strong anesthetic and hypnotic effects, but little effect on blood gases, ventilation, or the cardiovascular system. It has been proposed as an induction anesthetic (Stephenson et al., 2000). Etomidate shows low cytotoxicity, inhibits cell adhesion, and suppresses migration and invasion in A549 cells (Chu et al., 2019). In addition, using Etomidate for anesthesia has little effect on immune function in patients with LUAD (Liu et al., 2016). All these results indicate that lnc-eQTMs play essential roles in the development and treatment of LUAD and LUSC.

## Discussion

The associations between epigenetic modifications, such as aberrant DNA methylation and histone modifications, and lncRNA expression play a role in many kinds of diseases, including cancer. DNA methylation and lncRNA expression could influence each other. The regulation of DNA methylation by lncRNAs is an important mechanism that controls lncRNA expression during cancer progression and affects patient outcome. LncRNA HOTAIR was upregulated and promoted DNA methylation in the promoter region of PTEN, which is a tumor suppressor gene, and contributed to the dysfunction of PTEN in human laryngeal squamous cell cancer (Li et al., 2013). LncRNAs could also guide chromatin-modifying complexes to specific genomic loci and influence lncRNA expression. For example, PRC2, which is composed of methylase EZH2, SUZ12, and EED, is a major chromatin regulatory factor. PRC2 is involved in trimethylation of specific lysine residues (K9 and K27) in histone H3 (H3K9me3 and H3K27me3) (Tsai et al., 2010). Several studies have suggested that lncRNAs could be regulated by DNA methylation in cancers. For instance, the expression level of lncRNA CTC-276P9.1 was associated with the aberrant hypermethylation of regions around the transcription start site in esophageal cancer (Xiao et al., 2019). In the present study, we identified DMSs and their related lnc-eQTMs in LUAD and LUSC. We tried to explain the mechanism of lnc-eQTMs in diverse lung cancer subtypes. LUAD- and LUSC-specific lnc-eQTM networks were constructed based on absolute difference between PCCs of methylation levels and lncRNA expression. The selection of threshold is referred to our experience and published literature (Zhi et al., 2018). In future work, more choice and verification for absolute difference between PCCs would be provided.

LUAD and LUSC are the two main histological types of NSCLC. To classify the specific mechanisms underlying LUAD and LUSC, many studies have explored these mechanisms at diverse levels. Dong et al. (2020) comprehensively analyzed the aberrantly expressed profiles of lncRNAs and miRNAs with associated competing endogenous RNA network in LUAD and LUSC. Tian et al. (2020) assessed differences in mRNA and lncRNA expression between LUAD and LUSC. Genes and lncRNAs associated with cell junctions exhibit specific patterns in the two major histological subtypes. These studies all focused on the different mechanisms between LUAD and LUSC based on molecular expression profiles. In the present study, we validated that there are differences at the methylation level between LUAD and LUSC. In addition, LUAD and LUSC also share many common DMSs. Although some common lnc-eQTMs were found in LUAD and LUSC, the changed mechanisms were different, even opposite. These results indicate that LUAD and LUSC are caused by different dysfunctional pathways. Our work provided a standardized pipeline for identifying lnc-eQTMs in cancers and also provided 593 and 55 candidate markers for LUSC and LUAD, respectively. These mechanisms should be explored in more detail and validated in future works.

In summary, our work provides novel insights into the potential function of lnc-eQTMs in LUAD and LUSC. We identified DMSs and LUAD- and LUSC-specific lnc-eQTMs. The common lnc-eQTMs also showed different regulatory mechanisms between LUAD and LUSC and different possible clinical treatments. Some prognosis-associated lnc-eQTMs were also discovered in LUAD and LUSC based on an integrated computational approach. The functional and drug analyses also revealed the essential role of lnc-eQTMs in lung cancer. Overall, the investigation and identification of the specific lnc-eQTMs could contribute to our understanding of the epigenetic regulation in LUAD and LUSC by revealing functional eQTMs.

## Data Availability Statement

The datasets generated for this study can be found in the online repositories. The names of the repository/repositories and accession number(s) can be found in the article/Supplementary Material.

## Author Contributions

GW and SN conceived and designed the experiments. XW and YG analyzed the data and wrote this manuscript. JB, LD, and PZ collected the data. MC, LJ, and XS validated the method and data. All authors read and approved the final manuscript.

## Conflict of Interest

The authors declare that the research was conducted in the absence of any commercial or financial relationships that could be construed as a potential conflict of interest.

## Footnotes

1 https://bedtools.readthedocs.io/en/latest/content/overview.html

2 https://cytoscape.org/

3 http://amp.pharm.mssm.edu/Enrichr
