# Supplementary figures and images for: Identification of Potential Long Non-coding RNA Expression Quantitative Trait Methylations in Lung Adenocarcinoma and Lung Squamous Carcinoma
Source: Front Genet. 2020 Dec 9;11:602035. doi: 10.3389/fgene.2020.602035 (PMC7756030; doi:10.3389/fgene.2020.602035)

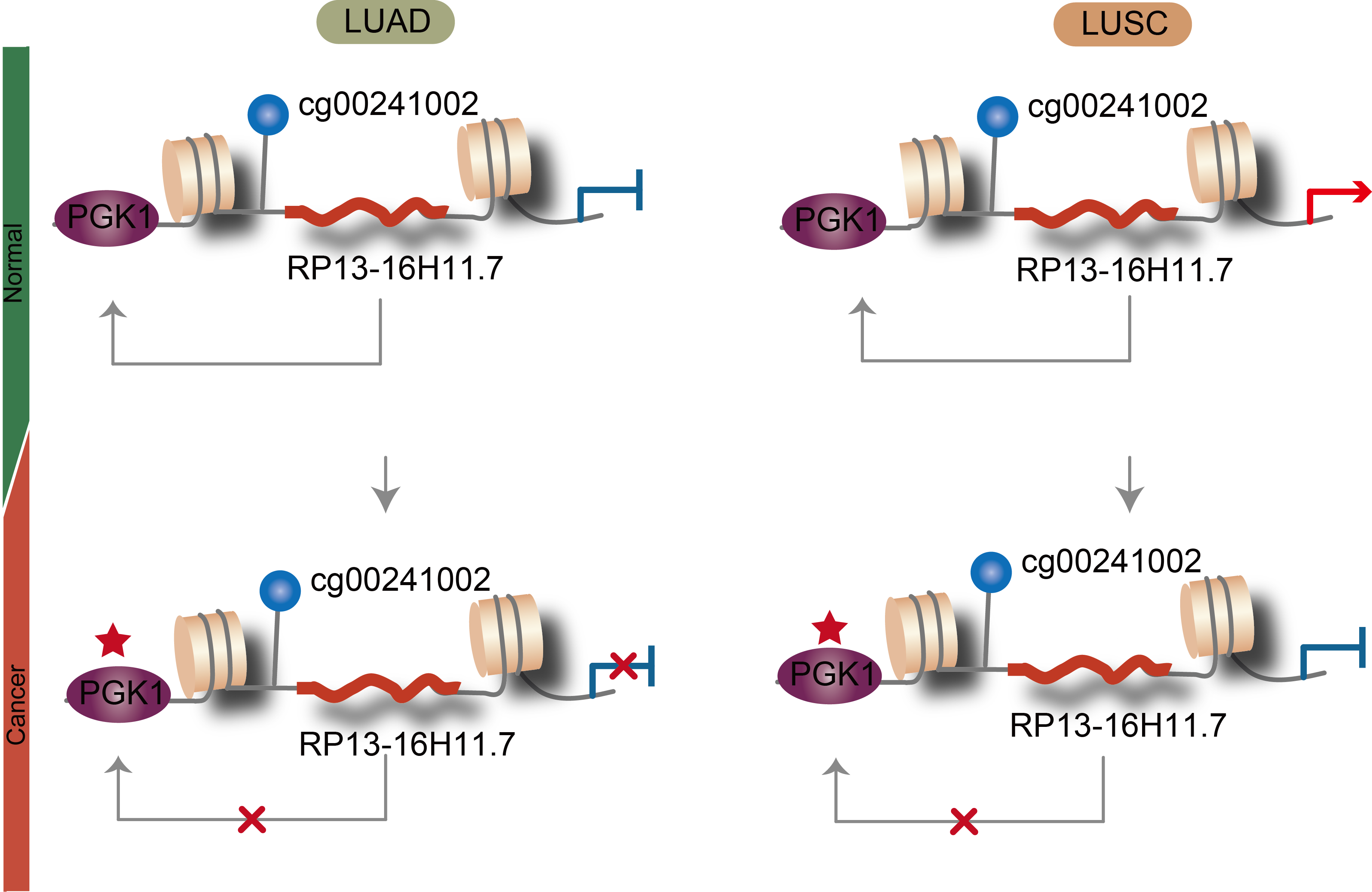

Supplement: Supplementary file 1 [file Image_1.TIF]
